# Retraction: Fluid–Structure Interaction-Based Biomechanical Perception Model for Tactile Sensing

**DOI:** 10.1371/journal.pone.0224189

**Published:** 2019-10-16

**Authors:** The PLOS ONE Editors

After publication of this article [1], the author notified the journal of several concerns about the reported study and, as such, we had the article re-assessed by a member of *PLOS ONE*’s Editorial Board. The following concerns were raised by the author and/or the Academic Editor:

- The experimental design was not sufficiently rigorous to address the study objectives and support the article’s conclusions. The author tested only one condition in the simulation, tested only one subject, did not test different kinds of static and dynamic load conditions, and did not assess the model’s performance using varied sensory corpuscle positions. Furthermore, questions were raised about the validity of the device used to simulate a finger in validation studies. Hence, the validity and generalizability of the model and the simulation results have been called into question, and the Academic Editor advised that the data presented are not sufficient to characterize the behavior of the device.
- Force value measurements were obtained from a dynamometer (newton) but conclusions based on pressure cannot be drawn as measurements were not taken using a manometer or pressure gauge (atm).
- The author did not distinguish between the haptic and tactile field in interpreting the results.
- According to the author, the experimental design incorporated a mistaken assumption about the consistent dimensions between the simulated finite element model and the real experimented object. Details of the finite element models and the dimensions of the haptic device are not sufficiently reported.
- Concerns were raised about reproducibility because the study is not clearly reported overall and the experimental design was not described in sufficient detail.

In light of the above issues, the *PLOS ONE* Editors retract the article due to concerns about the validity of the study’s experimental design and conclusions. Overall, the experiments conducted did not sufficiently validate the proposed model so as to meet the journal’s publication criteria for articles presenting novel methods or tools.

ZW did not comment on the final retraction decision and notice.
